# Development of a predictive model for radiation pneumonitis based on plasma exosomal miR-200b-5p

**DOI:** 10.3389/fonc.2025.1516348

**Published:** 2025-08-20

**Authors:** Shuwei Zhai, Yajun Zhu, Xiaoye Wang, Qingfeng Zhao, Xu Liang, Shuhao Que, Enhui Dai, Huaiyu Wang, Yuetong Li, Haihua Yang, Wei Feng

**Affiliations:** ^1^ Department of Radiation Oncology, Changzhou Jintan First People’s Hospital, Changzhou, China; ^2^ Department of Radiation Oncology, The Cancer Hospital of the University of Chinese Academy of Sciences (Zhejiang Cancer Hospital), Hangzhou, China; ^3^ Department of Radiation Oncology, The Affiliated Taizhou Hospital, Wenzhou Medical University, Taizhou, China

**Keywords:** exosomal miRNAs, radiation pneumonitis (RP), non-small cell lung cancer (NSCLC), predictive model, miR-200b-5p

## Abstract

**Objective:**

This study aims to explore the association between plasma exosomal miRNAs and the development of radiation pneumonitis (RP) in non-small cell lung cancer (NSCLC) patients who underwent radiotherapy, and develop a predictive model for symptomatic radiation pneumonitis (SRP) by integrating miRNA expression levels with clinical and dosimetric parameters.

**Methods:**

A total of 95 NSCLC patients, who were scheduled to receive definitive radiotherapy, were prospectively enrolled. Plasma exosomes were collected before the radiotherapy, and high-throughput sequencing followed by bioinformatics analysis was performed to identify the candidate miRNAs associated to SRP. Then, the expression levels of these miRNAs were validated using RT-qPCR. Afterwards, a predictive model for SRP was constructed using a nomogram, which combined the miRNA expression data with the clinical and dosimetric factors.

**Results:**

Among the 95 patients, 20 (21.10%) patients developed SRP. The high-throughput sequencing revealed 220 differentially expressed miRNAs. Among these miRNAs, 168 miRNAs were upregulated and 52 miRNAs were downregulated in SRP patients (*p*<0.05). The bioinformatics analysis identified miR-200b-5p as the key candidate miRNA. The univariate and multivariate analyses revealed that lung V5 (OR: 1.264, 95% CI: 1.042-1.532, *p*=0.018), mean lung dose (MLD; OR: 1.013, 95% CI: 1.004-1.023, *p*=0.006), and miR-200b-5p expression (OR: 0.144, 95% CI: 0.024-0.877, *p*=0.032) were the independent risk factors for SRP. The nomogram model that incorporated these factors achieved an area under the receiver operating characteristic curve (AUC) of 0.844, outperforming the individual factors alone (lung V5: 0.748, MLD: 0.760, and miR-200b-5p expression: 0.666).

**Conclusion:**

The combination of lung V5 >46.36%, MLD >1120 cGy, and miR-200b-5p expression <0.445 can be used to effectively predict the occurrence of SRP in locally advanced NSCLC patients. This model can aid in the early identification of patients at high risk for RP, allowing for personalized treatment adjustments and improved patient outcomes.

## 1 Introduction

Radiation therapy is a fundamental component in the treatment of locally advanced unresectable non-small cell lung cancer (NSCLC). Despite the advancements in radiotherapy techniques, radiation pneumonitis (RP) remain as a significant dose-limiting toxicity that poses a major challenge to achieving optimal tumor control, adversely impacting the quality of life of patients. Symptomatic radiation pneumonitis (SRP) (1, 2), particularly at grade 2 or higher (3), remains as a substantial clinical challenge, since this limits the radiotherapy dose required for effective tumor management, and can lead to serious complications in thoracic radiotherapy (4–6).

MicroRNAs (miRNAs), which is a class of non-coding RNA molecules, play a crucial role in the post-transcriptional regulation of gene expression (7, 8). Its involvement in radiation response has garnered considerable attention in recent years. Research has revealed that plasma miRNAs can serve as biomarkers for detecting radiation-induced damage in normal tissues, such as radiation esophagitis, brain injury, and cardiotoxicity (9, 10). Furthermore, miRNAs have been implicated in modulating the radiosensitivity of tumor cells, thereby influencing treatment outcomes (11, 12).

Most miRNAs in plasma are encapsulated within exosomes, which are the small extracellular vesicles that protect these from degradation. Exosomal miRNAs are particularly stable due to the protective lipid bilayer of exosomes, which shields these from enzymatic degradation in the bloodstream (13). This stability, along with its involvement in intercellular communication, makes exosomal miRNAs reliable biomarkers for various pathological conditions, including RP. Therefore, focusing on plasma exosomal miRNAs, rather than on total plasma miRNAs, would be a more accurate and reliable method for predicting RP.

Several studies have explored various predictors of RP, including clinical factors, such as performance status, lung function, tumor location, smoking history, and the presence of conditions (such as emphysema or interstitial lung disease) (14, 15). Furthermore, dosimetric factors, which include mean lung dose (MLD), gross tumor volume (GTV), and lung volumes that received specific radiation doses (lung V5, V10, V20 and V30), have been associated to RP incidence (16, 17). In addition, biological markers, such as interleukin (IL)-1, IL-6, and tumor necrosis factor-alpha (TNF-α), have been linked to the development of RP (18). However, despite these findings, the predictive power of these factors remain limited.

Given the need to improve the predictive accuracy for RP, the present study aimed to identify candidate plasma exosomal miRNAs with potential predictive value for SRP, and subsequently develop an integrated predictive model based on validated miRNA expression levels, clinical features, and dosimetric parameters. This model would significantly enhance the precision of RP prediction, facilitating better risk stratification and personalized treatment planning. Furthermore, this approach aligns with the broader goals of precision medicine, where the integration of individual genomic information with clinical and dosimetric parameters can lead to more tailored and effective therapeutic strategies. Ultimately, identifying reliable biomarkers, such as miR-200b-5p, would play a pivotal role in improving the management of RP, and enhancing patient outcomes in NSCLC treatment.

## 2 Materials and methods

### 2.1 Patient selection and ethics approval

**Figure 1:**
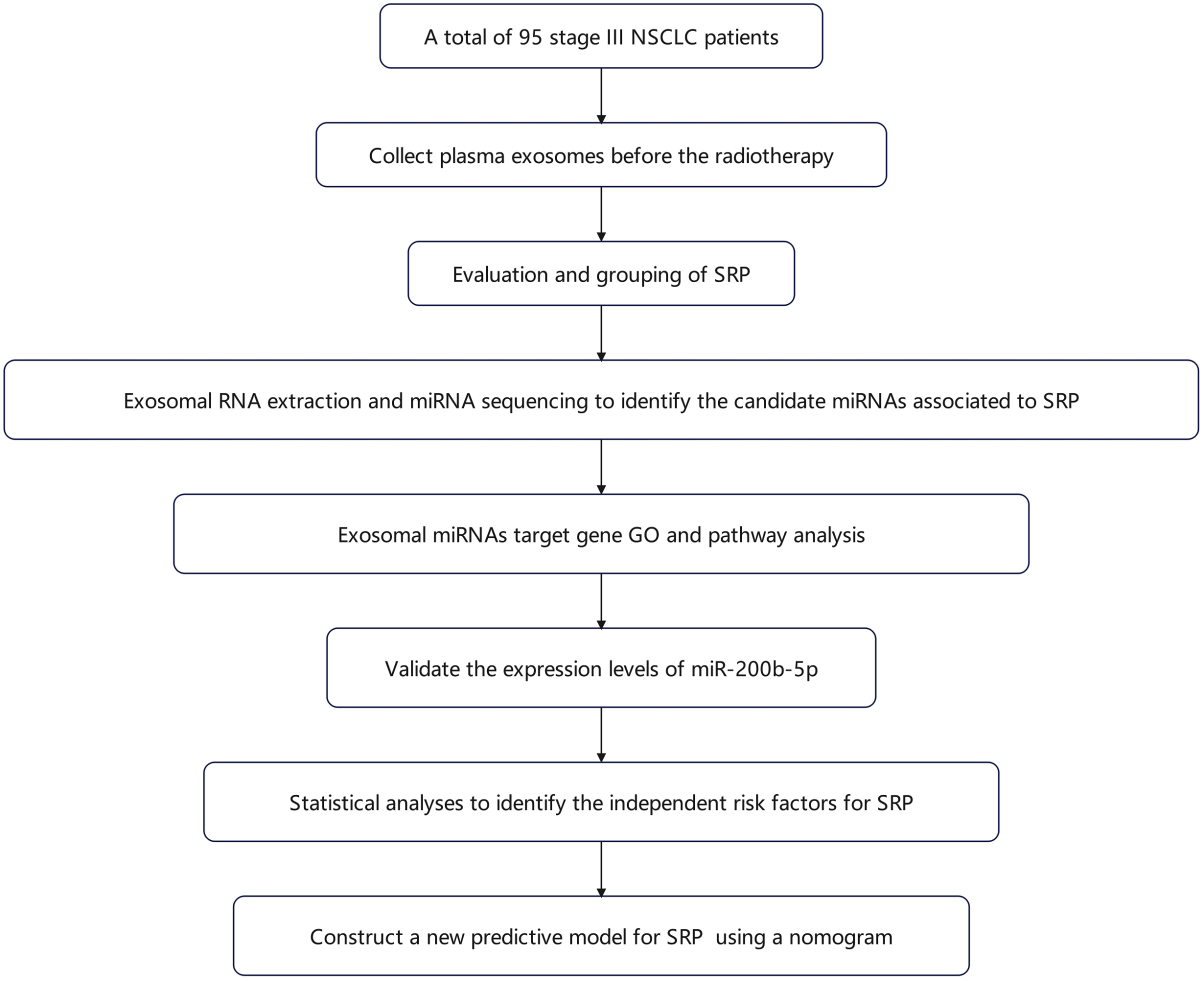
Flowchart for the study methodology. The schematic diagram outlines the sequence of procedures undertaken in the present study, which includes the following: patient recruitment, plasma exosome isolation and characterization, high-throughput miRNA sequencing and bioinformatic analysis, RT-qPCR validation of miR-200b-5p, and the construction of the predictive nomogram for symptomatic radiation pneumonitis.

A total of 95 NSCLC patients, who were treated with definitive radiotherapy at Zhejiang Cancer Hospital, between March 2022 and May 2023, were prospectively enrolled. Inclusion criteria: confirmed NSCLC, Eastern Clinical Oncology Group (ECOG) performance status ≤2, and clinical stage III disease. Exclusion criteria: patients with pulmonary infections during radiotherapy, patients with interruptions that exceeded two weeks, or patients with incomplete treatment. The ethics approval was obtained from the hospital’s ethics committee. The overall methodological workflow of the study, including patient enrollment, sample processing, and data analysis, is illustrated in **Figure 1**.

### 2.2 Data collection

The following comprehensive clinical and dosimetric data were collected: patient demographics, smoking history, performance status (PS) score, tumor characteristics, and treatment details. The key dosimetric parameters (lung V5 and V20, MLD, and mean heart dose [MHD]) were extracted from the dose-volume histograms (DVH).

### 2.3 Radiotherapy protocol

For patients who received radiotherapy, a total dose of 55-60 Gy was delivered using volumetric-modulated arc therapy (VMAT). The gross tumor volume (GTV) for the primary lung tumor was delineated on the computed tomography (CT) images using the lung window, while positive lymph nodes were contoured as GTVnd in the mediastinal window. A 6-8 mm margin was added to the GTV, and a 5 mm margin was added to the GTVnd, in order to form the clinical target volume (CTV), with adjustments made near the anatomical barriers. An additional 5 mm expansion was applied to both GTV and CTV, in order to generate the planning target volume (PTV). Organs at risk, which included the lungs, heart, spinal cord, and esophagus, were contoured following the RTOG 1106 guidelines, in order to ensure safe dose limits.

### 2.4 Evaluation and grouping of radiation pneumonitis in NSCLC patients

RP was systematically evaluated in NSCLC patients by chest CT scans conducted at the end of radiotherapy, and at 1, 3 and 6 months, post-treatment. The assessment was performed by a senior radiologist, in conjunction with a chief physician, who collaboratively reviewed the scans, and distinguished RP from other pulmonary conditions, such as infections, using both imaging and laboratory data. RP was classified according to the Common Terminology Criteria for Adverse Events (CTCAE v5.0), with SRP defined as grade 2 or higher. Based on these evaluations, the 95 patients were classified into non-SRP and SRP groups.

### 2.5 Exosome isolation and characterization

Plasma exosomes were isolated from 10 mL of blood collected from patients before radiotherapy. The blood samples were initially centrifuged at 3,700 rpm for 15 minutes at 4°C to separate the plasma. Then, the supernatant was carefully extracted and centrifuged again under the same conditions to ensure purity. Subsequently, 4 mL of plasma was diluted with phosphate buffered saline (PBS) to a final volume of 10 mL, and subjected to a series of differential centrifugation steps to isolate the exosomes (19, 20):

- Initial centrifugation: The plasma was centrifuged at 300 g for 10 minutes at 4°C to remove cellular debris. Then, the supernatant was collected and transferred to a new tube.
- Secondary centrifugation: The supernatant was further centrifuged at 2,000 g for 10 minutes at 4°C to eliminate larger vesicles and the remaining cell debris. Then, the supernatant was carefully collected.
- High-speed centrifugation: In order to pellet smaller vesicles, the supernatant was centrifuged at 12,000 g for 30 minutes at 4°C. Then, the resulting supernatant was transferred to ultracentrifuge tubes.
- Ultracentrifugation: The final step involved ultracentrifugation at 120,000 g for 70 minutes at 4°C. The resulting pellet, which contained the exosomes, was resuspended in PBS to form the exosome solution.

For characterization, the isolated exosomes were resuspended in RNase-free PBS, and placed onto a copper grid for 10 minutes. Then, the exosomes were stained with 2% phosphotungstic acid for 30 seconds, and allowed to air-dry in room temperature. The morphological assessment was conducted by transmission electron microscopy (TEM; Tecnai G2 Spirit Bio Twin, FEI, USA), which revealed the characteristic cup-shaped morphology and double-membrane structure of exosomes (21–23). In parallel, nanoparticle tracking analysis (NTA) was performed using a NanoSight instrument (Malvern Panalytical Ltd., UK) to determine the size distribution and concentration of exosomes in suspension.

### 2.6 Exosomal RNA extraction and miRNA sequencing

In order to ensure balanced clinical and dosimetric characteristics for downstream statistical analyses and miRNA expression profiling, five patients each from the SRP and non-SRP groups were matched based on age, gender, pathological type, tumor volume, and key dosimetric parameters, including lung V5, V20 and V40, and MLD.

Total RNA was extracted from exosomes using Trizol reagent. After adding 1 mL of Trizol to the sample, the mixture was homogenized and incubated for 15 minutes in room temperature. Then, chloroform (0.20 mL) was added, followed by vigorous shaking, and 15-minute incubation on ice. Afterwards, the mixture was centrifuged at 12,000 rpm for 15 minutes at 4°C, and the aqueous phase that contained the RNA was collected. Subsequently, the RNA was precipitated with isopropanol, washed with 75% ethanol, and resuspended in DEPC-treated water. Then, the RNA concentration was measured using a microplate reader.

For the miRNA sequencing, the miRNA libraries were constructed using the QIAseq miRNA Library Kit, in order to maximize the detection of miRNAs present in the samples. Unique Molecular Identifiers (UMIs) were incorporated during the library preparation to reduce polymerase chain reaction (PCR)-induced duplication errors, ensuring precise miRNA quantification. Next, the libraries were sequenced, and the sequencing reads were aligned to the miRBase database using the Bowtie software (version 1.2.2) for miRNA identification and quantification. Then, the UMI count for each miRNA was normalized using counts per million (CPM), and differential expression analysis was conducted using the edgeR package. Afterwards, the CPM values were log^2^-transformed, and miRNAs with a *p*-value of <0.05 were considered significantly and differentially expressed. The expression differences were visualized using scatter plots and clustering heatmaps, in order to highlight the variations in miRNA profiles between the different patient groups.

### 2.7 Exosomal miRNA target gene Gene Ontology and pathway analysis

The differentially expressed miRNAs identified in the present study were subjected to target gene prediction and annotation using the miRTarBase database. Then, the predicted target genes were further analyzed using the R packages for GO and pathway enrichment analysis. This bioinformatic approach was applied to uncover the key biological functions and signaling pathways involved in the regulation of genes associated to RP.

In order to identify the potential candidate miRNAs that might regulate RP, known RP markers were searched on disgenet.org. Then, the predicted target genes of the differentially expressed miRNAs were analyzed to determine its relevance to these markers. This analysis enabled the investigators to pinpoint miRNAs that were most likely involved in the regulation of RP-related pathways, providing insight into the molecular mechanisms underlying RP, and identifying targets for future therapeutic interventions.

### 2.8 Reverse-transcription quantitative polymerase chain reaction analysis of candidate miRNAs

After isolating the plasma exosomes through differential centrifugation, the total RNA was extracted using a rapid RNA extraction kit, according to manufacturer’s instructions. Then, the extracted RNA was reverse-transcribed into cDNA using a reverse transcription kit. For the RT-qPCR analysis, a reaction mixture was prepared in a 0.2 mL PCR tube (which contained 10.0 µL of TB Green, 0.8 µL of forward primer, 2.0 µL of reverse primer, and 6.8 µL of ddH_2_O), and miRNA-16 was used as the reference gene for normalization. Then, the relative expression level of miR-200b-5p was quantified using the 2^-ΔΔCt^ method. The primer sequences used for the RT-qPCR are listed in **Supplementary Table 1**. The RT-qPCR analysis was conducted to validate the expression levels of miR-200b-5p, which was identified as a candidate miRNA potentially involved in the regulation of RP (**Supplementary Table 1**).

### 2.9 Statistical analysis

The statistical analysis was conducted using SPSS version 25.0 and R software version 4.2.0. For paired patient data, Wilcoxon signed-rank test was employed to compare the groups. Univariate analysis was performed for each candidate variable. Categorical variables were compared using chi-square test or Fisher’s exact test, while continuous variables were analyzed using *t*-test or Mann-Whitney *U*-test, as appropriate. A significance level of *p*<0.05 was set for the univariate analysis. Spearman’s correlation analysis was conducted to account for the potential multicollinearity among variables. Variables with significant correlations were further analyzed using a multivariate logistic regression model, in order to identify the independent risk factors for SRP. Based on the results, a nomogram predictive model was constructed using the rms package in R. Then, the predictive accuracy of the nomogram was evaluated using receiver operating characteristic (ROC) curves. The model’s clinical utility was further assessed by calibration curves and decision curve analysis (DCA), in order to ensure its practical applicability in predicting SRP in clinical settings.

## 3 Results

### 3.1 Baseline characteristics

After the assessment and follow-up of the 95 patients who underwent radiotherapy for NSCLC, these patients were categorized into two groups, based on the occurrence of SRP: 75 (78.90%) patients were classified as non-SRP patients (non-SRP group), while 20 (21.10%) patients were classified as patients who developed SRP (SRP group). Among the patients in the SRP group, 16 (17.80%) patients had grade 2 RP, and four patients (4.20%) had grade 3 RP. No case of grade 4 or 5 RP was observed.

The cohort consisted of 78 male patients and 17 female patients, with a median age of 65 years old (range: 42-89 years old). The majority of these patients (67.40%) had a history of smoking, and squamous cell carcinoma was the predominant pathology (63.20%). For the treatment modalities, 26 (27.4%) patients received concurrent chemotherapy, and 62 (65.20%) patients underwent immunotherapy. The tumors were primarily located in the upper lung in 47 patients (49.50%), and the clinical stages of these patients were distributed, as follows: 22 patients were in stage IIIA, 48 patients were in stage IIIB, and 25 patients were in stage IIIC.

### 3.2 Isolation, characterization, and functional profiling of exosomal miRNAs associated to SRP

#### 3.2.1 Exosome isolation and miRNA profiling in the SRP and non-SRP groups

**Figure 2:**
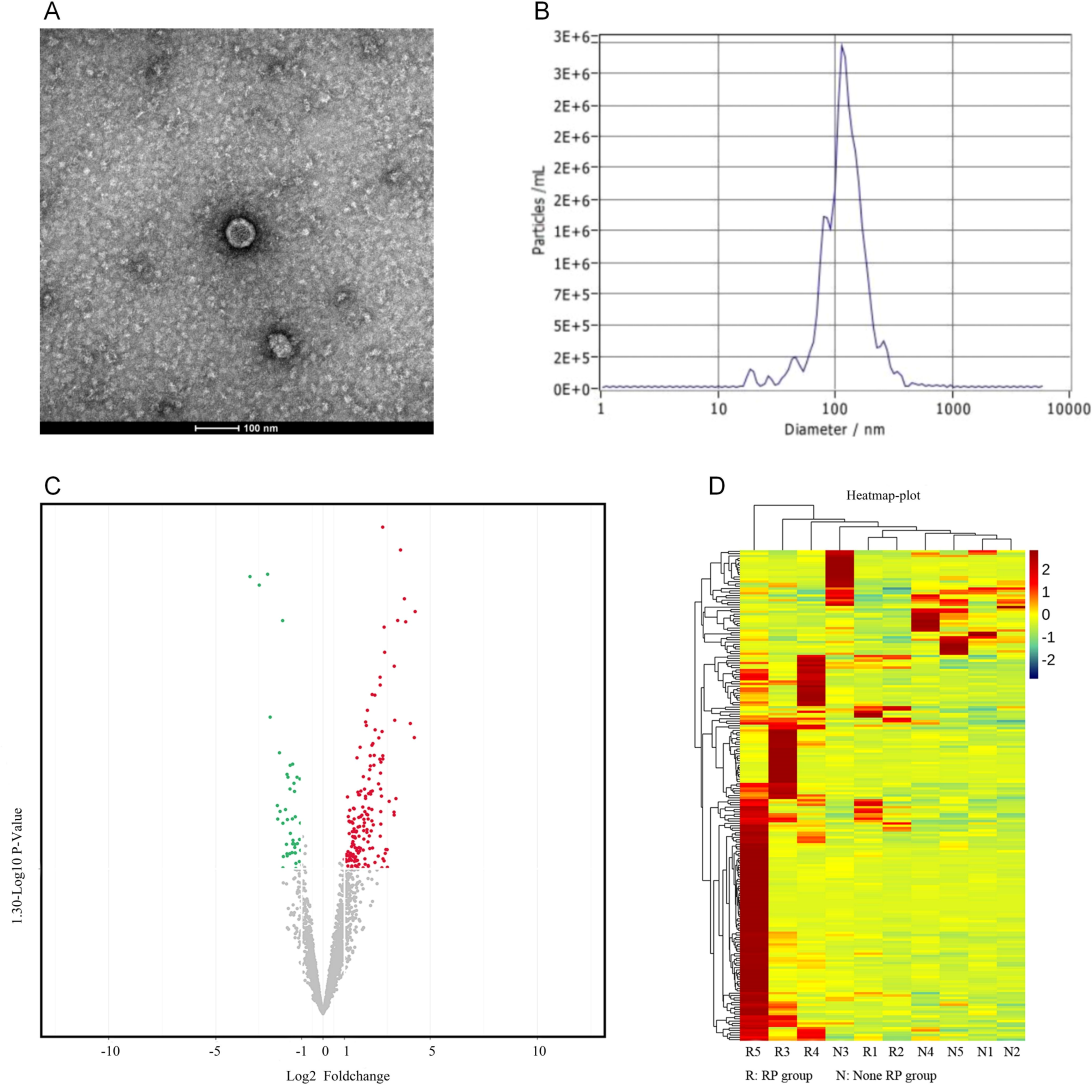
Exosome isolation and differential miRNA expression analysis. **(A)** The transmission electron microscopy (TEM) images show that the exosomes have a characteristic double-membrane, cup-shaped morphology. **(B)** The nanoparticle tracking analysis (NTA) indicated that the exosomes had a mean diameter of 114.10 ± 56.50 nm. **(C)** The volcano plot highlights the 220 miRNAs with differential expression: 168 miRNAs had an increased expression, and 52 miRNAs had a decreased expression in the SRP group, when compared to the non-SRP group (*p*<0.05). **(D)** The clustering heatmap presents the expression levels of differentially expressed miRNAs across all samples: red represents a higher expression, and green represents a lower expression.

The plasma exosomes were successfully isolated and characterized using TEM and NTA. The TEM images revealed that the exosomes exhibited a typical double-membrane cup-shaped morphology, which is consistent with the known characteristics of exosomes (**Figure 2A**). The NTA further confirmed that the size distribution of these exosomes had a mean diameter of 114.10 ± 56.50 nm, which falls within the expected range for exosomes (**Figure 2B**).

In order to identify the differentially expressed miRNAs associated to SRP, high-throughput sequencing was performed on a matched subgroup of 10 patients (five SRP and five non-SRP patients), who were selected to ensure comparability in age, tumor volume, lung V5, V20 and V40, and MLD (**Supplementary Table 2**). After exosome isolation, the expression profiles of miRNAs within these exosomes were analyzed to identify the potential biomarkers associated to SRP. The miRNA quantification revealed that 220 miRNAs presented with a differential expression between the SRP and non-SRP groups. Among these miRNAs, 168 miRNAs presented with an increased expression, and 52 miRNAs presented with a decreased expression in the SRP group (*p*<0.05). These results were visualized using a volcano plot, in which miRNAs that presented with significant changes in expression were highlighted (**Figure 2C**). Furthermore, a clustering heatmap was generated to illustrate the expression patterns of these differentially expressed miRNAs across all samples (red indicates higher expression levels and green indicates lower levels, **Figure 2D**).

#### 3.2.2 Functional enrichment analysis of target genes

The functional enrichment analysis of target genes of differentially expressed miRNAs was conducted by GO and Kyoto Encyclopedia of Genes and Genomes (KEGG) pathway analyses. The GO analysis was divided into three categories: molecular function, biological processes, and cellular components.

**Figure 3:**
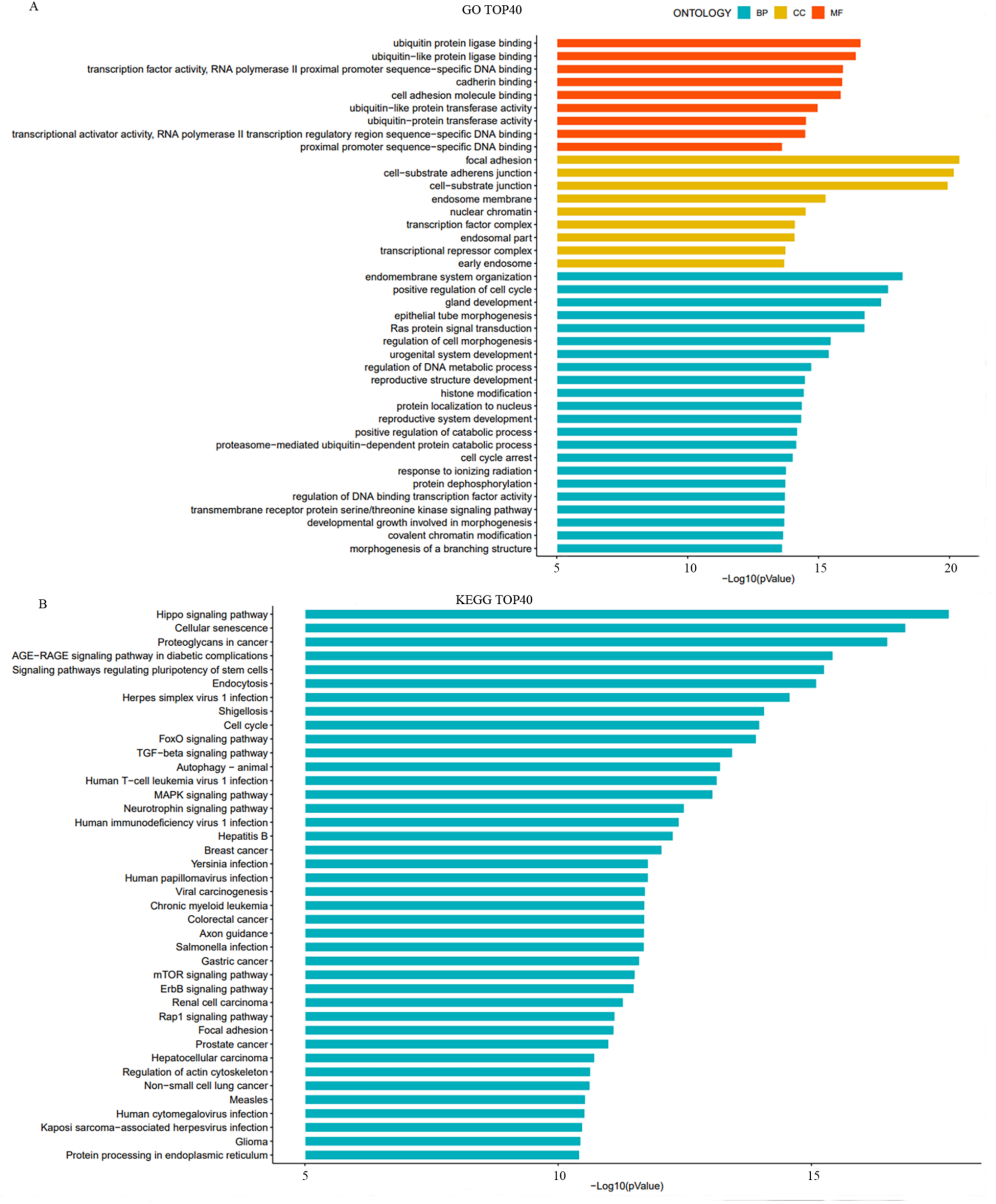
Gene Ontology (GO) and Kyoto Encyclopedia of Genes and Genomes (KEGG) analysis of target genes of differentially expressed miRNAs. **(A)** The bar charts illustrate the top 10 significantly enriched GO terms across three categories: biological processes (BP), cellular components (CC), and molecular function (MF). **(B)** The bar chart presents the top 40 significantly enriched signaling pathways identified by KEGG analysis.

In the molecular function category, the target genes were primarily enriched in cell adhesion molecule binding and ubiquitin-like protein ligase binding. For biological processes, the main enrichments were in the organization of the endomembrane system and positive regulation of the cell cycle. In the cellular components category, the target genes were associated to intracellular membranes, nuclear chromatin, and transcription factor complexes (**Figure 3A**).

The KEGG pathway analysis identified significant enrichment in several key signaling pathways, including the Hippo signaling pathway, FoxO signaling pathway, TGF-β signaling pathway, and Rap1 signaling pathway (**Figure 3B**). These pathways are known to play essential roles in cell growth, differentiation, and response to stress, which are critical in the context of cancer progression and treatment response.

#### 3.2.3 Identification of key miRNAs associated to SRP

**Figure 4:**
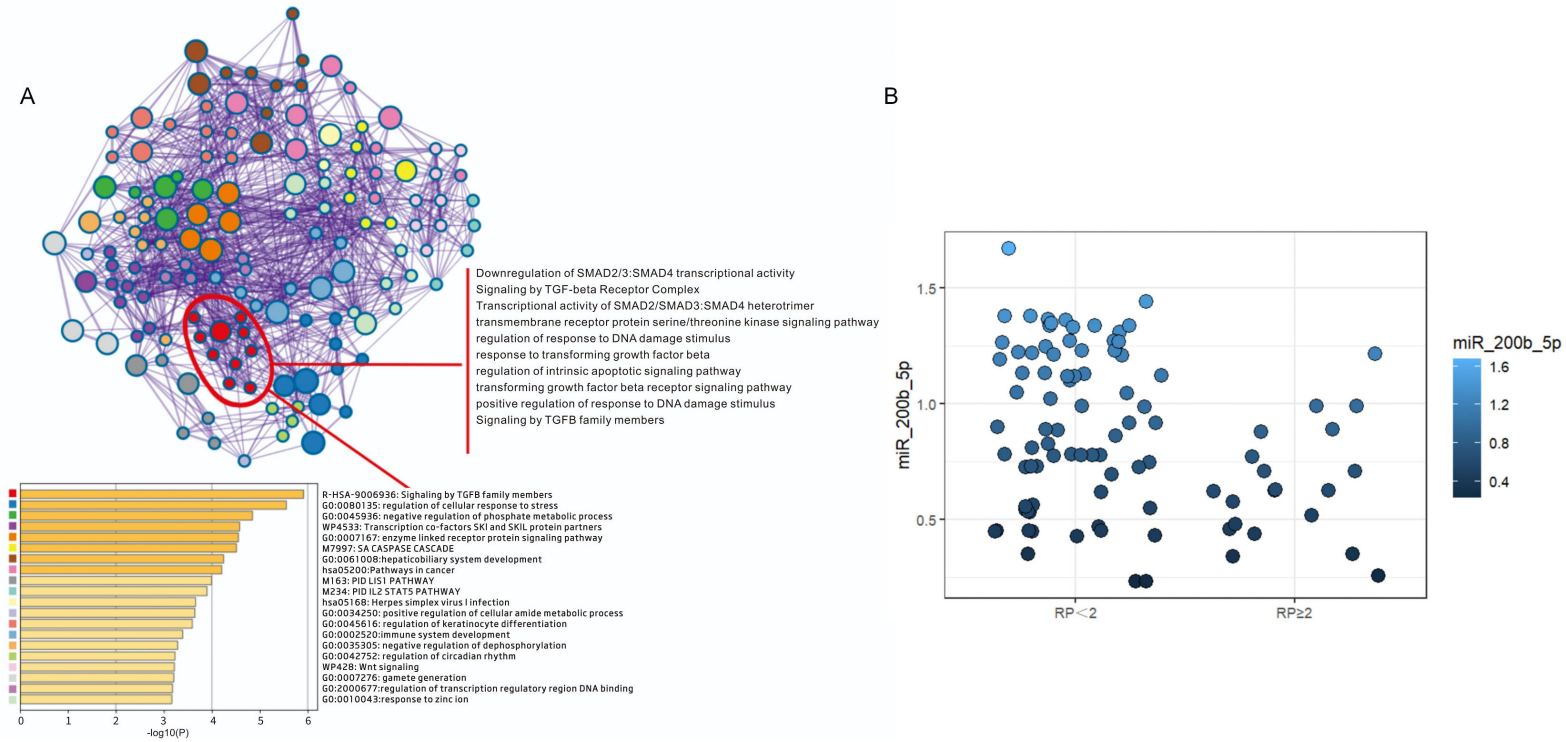
Functional enrichment and quantitative analysis of miR-200b-5p target genes. **(A)** The functional enrichment analysis revealed that the target genes of miR-200b-5p are predominantly involved in the regulation of the TGF-β signaling pathway. Specifically, miR-200b-5p targets key members of the TGF-β family, including TGF-β1, SMAD2, SMAD3 and SMAD4, playing a crucial role in modulating its translational regulation. **(B)** The reverse-transcription quantitative polymerase chain reaction analysis revealed the relative expression levels of miR-200b-5p in plasma exosomes obtained from symptomatic radiation pneumonitis (SRP) and non-SRP patients. The results indicate that the miR-200b-5p expression was significantly downregulated in the SRP group, when compared to the non-SRP group.

In order to identify the key miRNAs implicated in SRP, high-throughput sequencing was performed on a matched cohort of 10 patients (five SRP and five non-SRP patients). Fifteen miRNAs that exhibited a fold change of >8 between groups were shortlisted as candidate regulators for further analysis. The established markers for RP were identified using disgenet.org, which included ALOX5, CCL17, CCL22 and TGF-β. The target gene prediction for these 15 miRNAs revealed that miR-200b-5p was significantly enriched in the TGF-β signaling pathway, specifically targeting genes, such as TGF-β1, SMAD2, SMAD3 and SMAD4 (**Figure 4A**). Based on these findings, miR-200b-5p was selected as the key focus for further investigation.

### 3.3 Univariate and correlation analysis of dosimetric factors and risk factors

In order to elucidate the factors that contributed to the development of SRP, a univariate analysis of clinical characteristics was performed. The analysis revealed that concurrent chemotherapy (*x^2^ =* 4.711, *p*=0.030) and immunotherapy (*x^2^ =* 4.860, *p*=0.023) were significantly associated to the development of SRP. However, no significant differences were observed between the SRP and non-SRP groups, in terms of gender, age, smoking history, PS score, pathology type, clinical stage, or tumor location (**Table 1**).

**Table 1:** Univariate analysis of clinical variables.

Clinical factors	RP <2 *n*=75	RP ≥2 *n*=20	*x^2^*	*p*
Gender				
Male	62 (82.70%)	16 (80.00%)	0.076	0.782
Female	13 (17.30%)	4 (20.00%)		
Age				
≤65	37 (49.30%)	10 (50.00%)	0.063	0.832
>65	38 (50.70%)	10 (50.00%)		
Smoking history				
No	25 (33.30%)	6 (30.00%)	0.080	0.778
Yes	50 (66.70%)	14 (70.00%)		
PS score				
0-1	62 (82.70%)	15 (75.00%)	0.257	0.612
≥2	13 (17.30%)	5 (25.00%)		
Pathology type				
Squamous	48 (64.00%)	12 (60.00%)	0.109	0.742
Adenocarcinoma	27 (36.00%)	8 (40.00%)		
Tumor location				
Upper lobe	39 (52.00%)	8 (40.00%)	0.910	0.340
Middle/Lower lobe	36 (48.00%)	12 (60.00%)		
Clinical stage				
IIIA	20 (26.70%)	2 (4.60%)	3.698	0.157
IIIB	38 (50.70%)	10 (50.00%)		
IIIC	17 (22.70%)	8 (40.00%)		
Concurrent Chemotherapy				
Yes	19 (25.30%)	7 (35.00%)	4.711	0.030
No	56 (74.70%)	13 (65.00%)		
Immunotherapy				
Yes	47 (62.70%)	15 (75.00%)	4.860	0.023
No	28 (37.30%)	5 (25.00%)		

RP, radiation pneumonitis; PS, performance status.

The dosimetric parameters assessed in the univariate analysis included the following: PTV, total lung volume, lung dose-volume metrics (lung V5, V10, V20, and V40), MLD and MHD. These parameters were treated as continuous variables, and the normality tests indicated non-normal distributions across the groups. Accordingly, the Mann-Whitney *U*-test was applied to assess the differences between the SRP and non-SRP cohorts. The results revealed that lung V5, V10 and V20, and MLD were significantly associated to SRP occurrence (*p*<0.05, **Table 2**), suggesting that greater exposure to low- and intermediate-dose radiation may contribute to the pathogenesis of SRP.

**Table 2:** Univariate analysis of dosimetric variables.

Dosimetric factor	Non-SRP (*n*=75) Median (min, max)	SRP (*n*=20) Median (min, max)	*z*-value	*p*-value
PTV volume (cm^3^)	309.30 (94.90, 1,379.00)	439.10 (150.10, 795.20)	1.324	0.185
Total lung volume (cm³)	2984.00 (1,428.90, 4,630.80)	2,821.20 (1,717.70, 4,974.00)	0.233	0.816
Lung V5 (%)	40.40 (15.00, 57.50)	43.90 (36.20, 54.10)	2.297	0.022
Lung V10 (%)	30.20 (11.00, 43.10)	34.50 (28.20, 45.80)	2.361	0.018
Lung V20 (%)	19.30 (8.60, 28.30)	21.50 (18.20, 25.70)	2.484	0.013
Lung V30 (%)	13.00 (6.30, 21.20)	14.80 (10.00, 17.40)	0.977	0.067
Lung V40 (%)	7.80 (0.70, 16.00)	8.10 (5.30, 13.20)	1.374	0.169
MLD (cGy)	1,083.30 (507.00, 1,602.30)	1,274.20 (1,026.90, 1,511.20)	2.576	0.009
MHD (cGy)	1,010.30 (39.70, 2,461.20)	1,141.00 (198.00, 2,320.00)	0.685	0.152

SRP, symptomatic radiation pneumonitis; PTV, planning target volume; MLD, mean lung dose; MHD, mean heart dose.

Subsequently, Spearman’s correlation analysis was conducted to evaluate the relationship among the significant clinical and dosimetric factors identified in the univariate analysis before performing the multivariate analysis. The results indicated a strong correlation among several factors (**Supplementary Table 3**). Specifically, it was observed that a correlation coefficient (γ) of >0.70 can reduce the predictive accuracy of the model. In order to address the potential multicollinearity, and ensure model stability, lung V5, V10 and V20 were not simultaneously included in the multivariate analysis. Among these, lung V5 and MLD were selected for further analysis due to its lower correlation coefficient (γ=0.66), when compared to the other variables. Together with lung V5 and MLD, concurrent chemotherapy and pre-radiotherapy immunotherapy were included in the multivariate analysis to identify the independent risk factors for SRP.

The RT-qPCR analysis results revealed that the miR-200b-5p expression was significantly downregulated in the SRP group, when compared to the non-SRP group (*Z*=2.786, *p*=0.005), supporting its potential role as a predictive biomarker (**Figure 4B**, **Supplementary Table 4**). Furthermore, the Spearman’s correlation analysis indicated that the miR-200b-5p expression was not significantly correlated to other clinical factors, including lung V5, MLD, concurrent chemotherapy, and pre-radiotherapy immunotherapy (**Supplementary Table 5**), thereby supporting its inclusion in the multivariate analysis.

### 3.4 Multivariate logistic regression analysis

Finally, the multivariate logistic regression analysis was conducted, which incorporated lung V5, MLD, concurrent chemotherapy, pre-radiotherapy immunotherapy, and miR-200b-5p expression level. This analysis identified lung V5 (OR=1.265, *p*=0.018), MLD (OR=1.013, *p*=0.006), and reduced miR-200b-5p expression (OR=0.086, *p*=0.032) as independent risk factors for the development of RP (**Table 3**).

**Table 3:** Risk factors for radiation pneumonitis.

Risk factor	β	OR (95% CI)	*p*
V5	0.234	1.264 (1.042-1.532)	0.018
MLD	0.013	1.013 (1.004-1.023)	0.006
Chemotherapy	2.021	7.544 (1.070-32.205)	0.053
Immunotherapy	0.528	1.695 (1.006-9.669)	0.253
miR-200b-5p	-2.451	0.144 (0.024-0.877)	0.032

OR, odds ratio; 95% CI, 95% confidence interval; MLD, mean lung dose.

### 3.5 Development and validation of the nomogram prediction model

**Figure 5:**
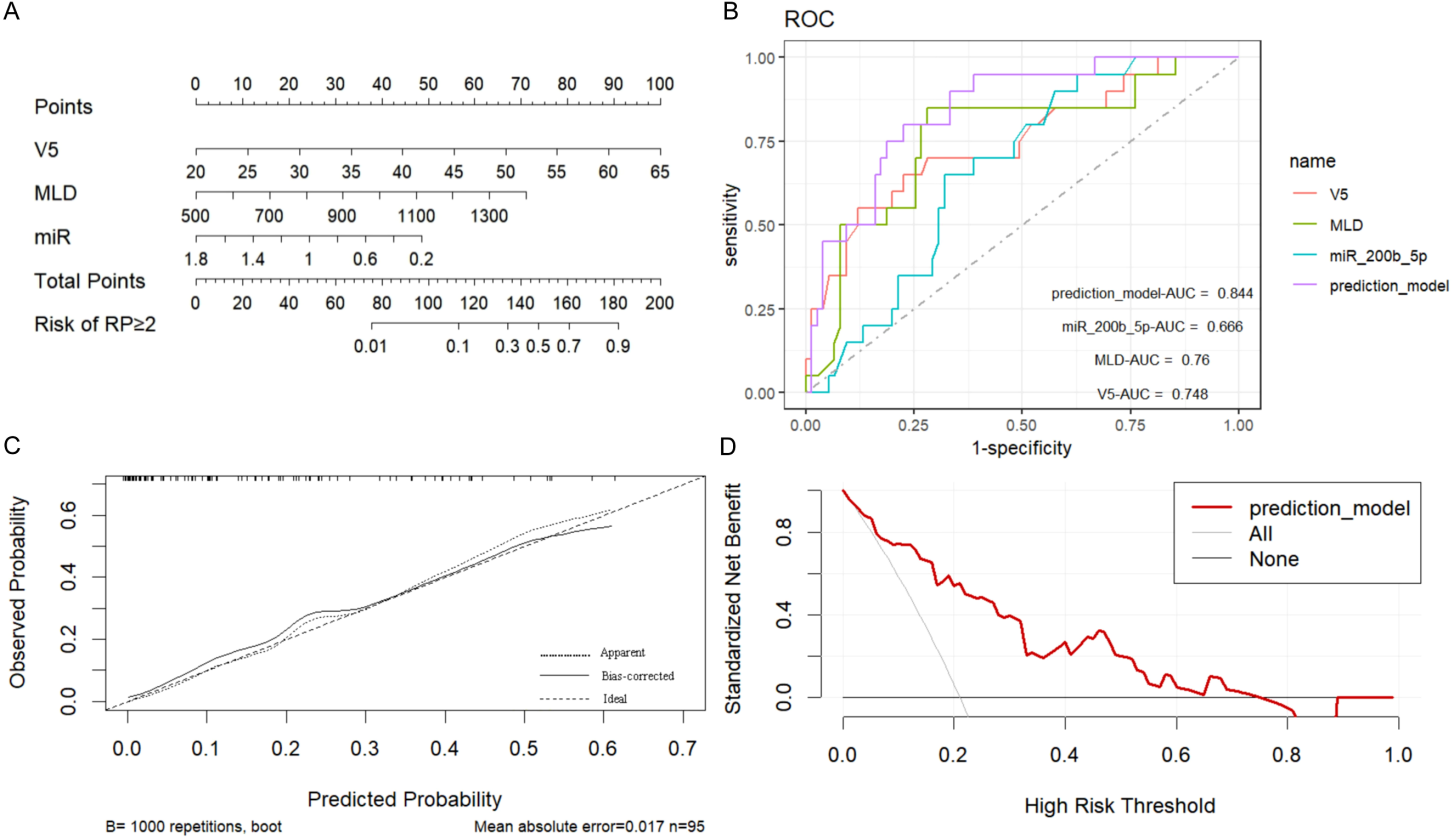
Construction and validation of the symptomatic radiation pneumonitis (SRP) prediction nomogram. **(A)** Nomogram prediction model for SRP: A nomogram was developed to predict the risk of SRP using the following independent risk factors identified in the present study: lung V5, mean lung dose (MLD), and low miR-200b-5p expression level. Each predictor was assigned a specific score on a points scale, and the total score corresponds to the probability of SRP occurrence at the bottom of the nomogram. **(B)** Receiver operating characteristic (ROC) curves for SRP prediction: The ROC curves demonstrate the discriminatory power of the nomogram, and the individual predictors (lung V5, MLD, and miR-200b-5p expression). The nomogram achieved an area under the receiver operating characteristic curve (AUC) of 0.844 (95% CI: 0.757-0.933), indicating superior predictive accuracy, when compared to the individual predictors alone. **(C)** Calibration curve of the nomogram: The calibration curve, which was derived from the 1000 bootstrap resampling iterations, presented a good agreement between the predicted and observed probabilities of SRP, confirming the nomogram’s reliability. **(D)** Clinical decision curve analysis: The decision curve analysis indicated that the nomogram provides a significant net benefit, when compared to the “treat all” or “treat none” strategies, particularly when the probability of SRP ranged between 0.10 and 0.70, highlighting the nomogram’s clinical utility.

The present study identified lung V5, MLD, and low miR-200b-5p expression level as independent risk factors for the development of acute RP. Based on these factors, a prediction model was constructed using the rms package in R (version 4.2.0), and a nomogram was generated (**Figure 5A**). In the nomogram, each predictor was assigned a score on a points scale at the top. By summing up the scores, the total score was calculated, which corresponded to the probability of developing SRP at the bottom of the nomogram.

The discriminatory power of the prediction model was evaluated using the area under the receiver operating characteristic curve (AUC). The model demonstrated a strong ability to distinguish between patients with and without SRP, with an AUC of 0.844 (95% CI: 0.757-0.933), and this was significantly higher than the AUCs for the individual predictors (lung V5 AUC: 0.748, 95% CI: 0.619-0.877; MLD AUC: 0.760, 95% CI: 0.634-0.885; miR-200b-5p expression AUC: 0.666, 95% CI: 0.551-0.781). The optimal cut-off value for lung V5, MLD and miR-200b-5p expression was 46.36%, 1,119.2 cGy and 0.445, respectively. The nomogram achieved high predictive efficiency, with a sensitivity of 81.30% and a specificity of 82.00% (**Figure 5B**, **Table 4**).

**Table 4:** ROC curve parameters.

Variables	AUC (95% CI)	Cut-off value	Sensitivity	Specificity
V5	0.748 (0.619-0.877)	46.355	0.880	0.560
MLD	0.760 (0.634-0.885)	1,119.200	0.920	0.761
miR-200b-5p	0.666 (0.551-0.781)	0.445	0.867	0.596
Nomogram	0.844 (0.757-0.933)	–	0.813	0.820

ROC curve, receiver operating characteristic curve; AUC, area under the receiver operating characteristic curve; 95% CI, 95% confidence interval; MLD, mean lung dose.

In addition, the internal validation of the model was performed using 1000 bootstrap resampling iterations, resulting in a calibration curve that demonstrated a good agreement between the predicted and observed probabilities of RP (**Figure 5C**). The DCA indicated that the net benefit of applying the nomogram was significantly higher, when compared to the “treat all” or “treat none” strategies, when the probability of RP was between 0.10 and 0.70, suggesting that the nomogram has good clinical utility (**Figure 5D**).

## 4 Discussion

The present findings demonstrated that lung V5 and MLD are significant independent risk factors for SRP, alongside the miR-200b-5p expression. Previous studies have reported that lung V5, as an indicator of low-dose radiation exposure, is closely associated to increased risk of RP by reflecting regional heterogeneity in lung inflammation. MLD has similarly been linked to the cumulative burden of radiation dose and its subsequent inflammatory response. This aligns with prior observations that lung dosimetric factors, including V5 and MLD, are critical determinants of lung toxicity in radiotherapy. Collectively, these data suggest that the integration of the miR-200b-5p expression with established dosimetric factors can enhance the predictive accuracy for SRP, offering a more nuanced approach to risk stratification and personalized radiotherapy planning.

In the present study, several clinical factors, including age, gender, smoking history, tumor histology, tumor site, and tumor stage, did not reach statistical significance in the univariate analysis. The results indicated that these variables had no significant impact on the development of SRP. Notably, the present analysis identified lung V5 as a significant independent risk factor for SRP. Similar findings have been reported in previous studies. For instance, lung V5 has been recognized as a predictor of SRP following radiotherapy in patients with lung cancer (24), and as a prognostic indicator of radiation-induced lung injury in patients with extensive-stage small cell lung cancer (25). Furthermore, in patients with mediastinal lymphoma and esophageal cancer undergoing radiotherapy, lung V5 has similarly been demonstrated as a predictor of RP (26). These observations collectively suggest that lung V5 may play a pivotal role in the pathogenesis of RP.

The significance of MLD in predicting RP has been well-established. Wang et al. reported that the incidence of acute RP was 13% in patients with MLD ≤16 Gy, when compared to 36% in patients with MLD exceeding 16 Gy. Claude et al. similarly confirmed that MLD is an independent risk factor for acute RP, with a predictive threshold of 13 Gy (27). Borst et al. (28) examined the relationship between MLD and the incidence of RP following stereotactic body radiotherapy, calculating MLD in the normalized total dose form using the linear-quadratic model with an α/β ratio of 3. They identified a significant dose-response relationship between RP and MLD. In a retrospective analysis of 400 lung cancer patients, Kwa et al. (29) reported a significant correlation between MLD and RP grade ≥2. In the present study, the optimal predictive value of MLD for acute RP, as determined by the ROC analysis, was 11.94 Gy, aligning closely with the thresholds identified in previous reports.

Exosomes play a critical role in intercellular communication by carrying essential biomolecules, such as DNA, mRNA, miRNA, lncRNA and proteins, between cells. These vesicles are pivotal in processes, such as immune response, tissue repair, and tumor progression (30). For patients who underwent radiotherapy, exosomes carry and transmit key signaling molecules that can induce responses in neighboring cells, including oxidative stress, the release of inflammatory cytokines, and immune cell recruitment. All of these contribute to the development of RP (31–33). Previous studies have revealed that the release of exosomes from bronchial epithelial cells increase four-fold after radiation exposure, along with the upregulation of pro-inflammatory cytokines (34). For example, in a mouse model, mesenchymal stem cell-derived exosomes that carry miR-466f-3p were shown to target c-MET, reduce inflammation, and prevent radiation-induced lung injury (34). These findings underscore the potential role of exosomes, in both the pathogenesis and treatment of RP.

In the present study, the differential expression of 220 miRNAs was identified between SRP and non-SRP patients, with miR-200b-5p being significantly downregulated in SRP patients. The eight-fold decrease in miR-200b-5p expression in SRP patients, when compared to non-SRP patients, suggest its crucial role in the development of RP. The target gene prediction analysis indicated that miR-200b-5p is primarily involved in the TGF-β signaling pathway, which is widely recognized as a key mediator of RP (35). TGF-β is a potent pro-fibrotic and pro-inflammatory factor that promotes fibroblast proliferation, collagen synthesis, and immune cell recruitment, thereby exacerbating RP (36). Thus, miR-200b-5p may play a vital role in modulating RP through the regulation of the TGF-β pathway.

Over the past two decades, the miR-200 family has been established as critical regulators of various physiological and pathological processes, particularly in maintaining epithelial homeostasis and inhibiting fibrosis (36, 37). Studies have demonstrated that miR-200 family members can reduce radiation-induced tissue damage by inhibiting NF-κB and Smad2 activation, thereby decreasing the production of pro-inflammatory cytokines. For instance, in radiation-induced oral mucositis, the miR-200 family alleviates inflammation by suppressing cytokines, such as TGF-β, TNF-α and IL-1α (38). Similarly, the overexpression of miR-200b inhibits fibrosis markers, such as vimentin and α-SMA, while increasing E-cadherin levels, indicating its protective role against pulmonary inflammation and fibrosis (39, 40). These studies provide strong evidence that miR-200b-5p can be a key modulator in the development and progression of RP.

The present study identified that the miR-200b-5p expression level, lung V5, and MLD are independent risk factors for RP. The predictive model constructed using these three factors achieved a high AUC of 0.844, significantly outperforming models that rely on individual parameters alone. This indicates that low miR-200b-5p expression is strongly associated to higher risk of RP, and that combining the miR-200b-5p expression level with dosimetric factors, such as lung V5 and MLD, can enhance the accuracy of RP risk prediction. This model has high sensitivity (81.30%) and specificity (82.00%), and provides a valuable tool for personalized RP risk assessment, guiding clinicians in optimizing radiotherapy plans to minimize the occurrence of RP, and thereby improving patient outcomes and quality of life. Importantly, the present findings are consistent with the findings of recent studies that developed comprehensive RP predictive models. Wang et al. created a nomogram that incorporated clinicopathologic, dosimetric, and biological factors, such as MLD and the systemic immune inflammation index, showing high discrimination (C-index = 0.852), and stratified patients into risk groups (41). Similarly, Tang et al. developed a nomogram that combined severe acute radiation-induced esophagitis (SARE), mean esophagus dose, and ipsilateral mean lung dose, further highlighting the predictive value of integrating clinical and dosimetric factors (42). These findings, alongside the present findings, underscore the importance of incorporating both molecular and dosimetric markers into risk prediction models for RP. Nevertheless, caution is warranted. The relatively modest sample size (*n*=95) and absence of external validation constrained the generalizability of these findings. In addition, the absence of significant associations for established clinical factors, such as age, smoking status, and ECOG performance status, within the present cohort may reflect sample size constraints and patient selection criteria. Future investigations with larger, independent cohorts are essential to validate the predictive capacity of this model, and confirm its clinical applicability.

## 5 Conclusions

Despite these significant findings, the present study had several limitations. The small sample size may have introduced bias. Thus, future studies with larger cohorts are needed to validate these results. In addition, although the predictive model was internally validated, external validation in independent cohorts is necessary to confirm its clinical utility. Furthermore, subsequent studies should focus in elucidating the downstream target genes and signaling pathways regulated by miR-200b-5p, which would help to better understand the biological mechanisms that drive RP. These studies would lead to the development of new strategies for the prevention and treatment of RP.

## Data Availability

The raw data supporting the conclusions of this article will be made available by the authors, without undue reservation.
