# Supplementary material for: Development of a predictive model for radiation pneumonitis based on plasma exosomal miR-200b-5p
Source: Front Oncol. 2025 Aug 20;15:1516348. doi: 10.3389/fonc.2025.1516348 (PMC12405333; doi:10.3389/fonc.2025.1516348)
Supplement: Supplementary file 1 [file Table1.doc]

**Supplementary** table 1. PCR primer sequences.

| **Name** |  | **Sequence** |
| --- | --- | --- |
| miR-16 | Sense | AAGCACCTAGCAGCACGTAAATA |
|  | Antisense | TATGGTTTTGACGACTGTGTGAT |
| miR-200b-5p | Sense | GCCCATCTTACTGGGCAGC |
|  | Antisense | GTGCAGGGTCCGAGGT |

**Note:** PCR, polymerase chain reaction

**Supplementary table 2. Baseline characteristics of the 10 paired patients.**

|  | **non-SRP (*n*=5)**  **Median (Min, Max)** | **SRP (*n*=5)**  **Median (Min, Max)** | **Z** | ***P*** |
| --- | --- | --- | --- | --- |
| Age | 67 (58, 72) | 65 (53, 74) | -0.67 | 0.500 |
| Tumor volume | 182.30 (75.10, 230.50) | 120.30 (95.10, 210.60) | -0.14 | 0.893 |
| V5 | 40.40 (30.70, 50.50) | 40.20 (35.20, 49.70) | -0.14 | 0.893 |
| V20 | 18.60 (16.30, 25.60) | 20.10 (19.80, 28.20) | -1.49 | 0.136 |
| V40 | 6.10 (4.00, 8.30) | 6.30 (5.30, 9.10) | -0.41 | 0.683 |
| MLD | 12.40 (10.00, 13.80) | 11.40 (9.70, 17.20) | -0.41 | 0.686 |

**Note:** MLD, mean lung dose

**Supplementary table 3**. Correlation coefficients of clinical and dosimetric factors.

|  | **V5** | **V10** | **V20** | **MLD** | **Chemotherapy** | **Immunotherapy** |
| --- | --- | --- | --- | --- | --- | --- |
| V5 | 1.000 | 0.887 | 0.755 | 0.660 | 0.070 | -0.182 |
| V10 | 0.887 | 1.000 | 0.750 | 0.703 | 0.050 | -0.260 |
| V20 | 0.755 | 0.750 | 1.000 | 0.842 | 0.090 | -0.295 |
| MLD | 0.660 | 0.703 | 0.842 | 1.000 | 0.045 | -0.232 |
| Chemotherapy | 0.070 | 0.050 | 0.090 | 0.045 | 1.000 | 0.198 |
| Immunotherapy | -0.182 | -0.260 | -0.295 | -0.232 | 0.198 | 1.000 |

**Note:** MLD, mean lung dose

**Supplementary table 4**. Relative expression levels of plasma exosomal miR-200b-5p in the 95 patients.

|  | **RP <2** | **RP ≥2** | ***z*-value** | ***p*-value** |
| --- | --- | --- | --- | --- |
|  | *n*=75 | *n*=20 |  |  |
| miR-200b-5p | 0.80 (0.23, 1.67) | 0.53 (0.16, 1.22) | 2.786 | 0.005 |

**Note:** RP, radiation pneumonitis

**Supplementary table 5**. Correlation between the miR-200b-5p expression level and clinical dosimetric factors.

|  | **V5** | **MLD** | **Chemotherapy** | **Immunotherapy** |
| --- | --- | --- | --- | --- |
| miR-200b-5p | -0.160 | -0.062 | 0.178 | 0.167 |
| *p*-value | 0.376 | 0.583 | 0.103 | 0.133 |

**Note:** MLD, mean lung dose
